# A Text-Book of the Practice of Medicine

**Published:** 1898-09

**Authors:** 


**A Text-Book of the Practice of Medicine.** By James M. Anders, M.D. One Volume in two. Pp. 1287. London: The Rebman Publishing Co. (Ltd.). Philadelphia: W. B. Saunders. 1898.

Of the making of text-books there appears to be no end: of late we have had quite a stream of guides in the domain of medicine. Osler, Allbutt, and Loomis and Thompson are now followed by Anders, whose two volumes in the now well-known blue covers form a very handsome ornament to the library table or book-shelf.

The aim of this new comer is to present a continuous story of each affection, including the pathology, the etiology, the clinical symptoms and the morbid lesions. A special feature of the work is the number of diagnostic tables which are found in it; there are no less than fifty-six of these, and the greater number are original. We notice a few formulæ which are introduced into the text, the metric equivalent of the dose being placed in parentheses: they are such as have been shown by large experience to be possessed of real therapeutic value. We have a great dislike to rigid formulæ, and to books which give pages of them together, and we quite sympathise with the author in his remark that the practising physician should form a therapeutic judgment for himself: he should be competent to devise for each patient the kind of drug or combination of drugs which is most suitable to the individual case. "Preference has been given to the modern orthography and terminology, not only because it is more euphonious, but also because of its adoption by the standard lexicographers." We have not observed any indications of the individual who makes his own spelling.

Any text-book must of necessity be much indebted to current medical literature, and one text-book must have a great family likeness to others of its kind; but this one has an individuality of its own, and we venture to think that its style is somewhat in advance of its predecessors: there is evidence in its pages of much personal observation, extending over a period of two decades, and derived from both hospital and private practice.

As in other text-books, this one begins with the consideration of infectious diseases, and of these typhoid fever takes the first place. Whilst the bacterium of Eberth is acknowledged to be the specific cause of the disease, it is pointed out that "the real poison of typhoid fever must be a chemical substance secreted by the bacillus—*typho-toxin*—and Brieger has extracted the latter agent, finding that it produces the fever, nervous symptoms, and the other manifestations characteristic of the affection." The bacteriological test of Widal, as simplified by Johnston of Montreal, is described, and it is remarked that "if this specific reaction is not obtainable in a case sick over a week, typhoid fever may be excluded. It is to be remembered, however, that in persons who have had typhoid fever within ten years the reaction may take place." The author has not specified the degree of dilution of the serum, or the time required for the characteristic clumping, and hence, perhaps, he also does not place the bacteriological test as one of the most trustworthy features of equal value with the step-ladder temperature curve, the continued type of the fastigium, the decline by lysis, the enlarged spleen, and the rose-coloured spots. He places the diazo-reaction of Ehrlich on an even lower level as regards utility in diagnosis; for, although he finds the reaction to be rarely absent after the eighth or ninth day, it is unfortunately also present in acute phthisis, meningitis, measles, and other infections attended with fever.

We hold to the view that both tests, the Widal and the Ehrlich, are of more value in the differential diagnosis from meningitis and acute tuberculosis than this and other text-books indicate: taken in conjunction with other clinical evidence, they give material aid in spite of the admitted fact that either test taken by itself is open to fallacy. We have recently learnt to rely on the Widal reaction as confirmatory of diagnosis made on other grounds, much in the same way as the finding of bacilli in the sputum confirms our diagnosis of phthisis made on the more fallacious physical signs; and just as no report on a case of phthisis is complete without an examination of sputum, so no report on a case of typhoid fever is complete without an examination of the serum.

It is gratifying to observe that the author has had no occasion to mention influenza as a probable or even possible cause of error in the diagnosis of typhoid fever. Dr. Anders gives careful instructions as to the disinfection of all the excreta, and he believes that the demands of hygiene cannot be fully satisfied without complete isolation of the patient on the same principles as those governing the treatment of other infectious diseases. The details of treatment do not materially differ from those commonly in use: an appropriate liquid diet and hydrotherapeutic methods are of course the chief points insisted on; as an intestinal antiseptic he prefers salol, of the internal antipyretics he prefers phenacetin. This article on typhoid fever is one of the most concise and withal as complete as any with which we are familiar: its arrangement is good, and its conclusions are clear.

The section on typhoid fever is a good illustration of the contents of the book, and we have no hesitation in commending the author's and the publishers' work as worthy of the highest praise.

